# The APSY-SED study: protocol of an observational, longitudinal, mixed methods and multicenter study exploring the psychological adjustment of relatives and healthcare providers of patients with cancer with continuous deep sedation until death

**DOI:** 10.1186/s12904-022-01106-z

**Published:** 2022-12-05

**Authors:** L. Fasse, N. Roche, C. Flahault, M. Garrouste-Orgeas, M. Ximenes, A. Pages, A. Evin, S. Dauchy, F. Scotte, JB. Le Provost, F. Blot, C. Mateus

**Affiliations:** 1 DIOPP, Gustave Roussy Hospital, Villejuif, France; 2 Institut de Psychologie Laboratoire de Psychopathologie et Processus de Santé, Université Paris Cité, 71 avenue E. Vaillant, F-92100 Boulogne- Billancourt, France; 3 IAME, INSERM, Université de Paris, F-75018 Paris, France; 4 Palliative Care unit, Reuilly Diaconesses Fondation, Rueil Malmaison, France; 5 Medical unit, French British Hospital, Levallois-Perret, France; 6 Maison Médicale Marie Galène, Bordeaux, France; 7 Biostatistical Unit, Gustave Roussy Hospital, Villejuif, France; 8 Palliative Care unit, CHU, Nantes, France; 9 DMU Psychiatry and Addictology, AP-HP.Centre, Université de Paris, Paris, France

**Keywords:** Continuous deep sedation until death, cancer, Palliative care, Psychological adjustment, bereavement/grief, Healthcare providers, Relatives

## Abstract

**Background:**

Since 2016, France is the only country in the World where continuous deep sedation until death (CDSUD) is regulated by law. CDSUD serves as a response to refractory suffering in palliative situations where the patients’ death is expected to occur in the following hours or days. Little is known on the psychological adjustment surrounding a CDSUD procedure for healthcare providers (HCPs) and relatives. Our study aims to gather qualitative and quantitative data on the specific processes behind the psychological adjustment of both relatives and HCPs, after the administration of CDSUD for patients with cancer.

**Methods:**

The APSY-SED study is a prospective, longitudinal, mixed-methods and multicenter study. Recruitment will involve any French-speaking adult cancer patient for who a CDSUD is discussed, their relatives and HCPs. We plan to include 150 patients, 150 relatives, and 50 HCPs. The evaluation criteria of this research are: 1/ Primary criterion: Psychological adjustment of relatives and HCPs 6 and 13 months after the death of the patient with cancer (psychological adjustment = intensity of anxiety, depression and grief reactions, CDSUD-related distress, job satisfaction, Professional Stress and Professional experience). Secondary criteria: a)occurrence of wish for a CDSUD in patients in palliative phase; b)occurrence of wish for hastened death in patients in palliative phase; c)potential predictors of adjustment assessed after the discussion concerning CDSUD as an option and before the setting of the CDSUD; d) Thematic analysis and narrative account of meaning-making process concerning the grief experience.

**Discussion:**

The APSY-SED study will be the first to investigate the psychological adjustment of HCPs and relatives in the context of a CDSUD procedure implemented according to French law. Gathering data on the grief process for relatives can help understand bereavement after CDSUD, and participate in the elaboration of specific tailored interventions to support HCPs and relatives. Empirical findings on CDSUD among patients with cancer in France could be compared with existing data in other countries and with results related to other medical fields where CDSUD is also conducted.

**Trial registration:**

This protocol received the National Registration Number: ID-RCB2021-A03042-39 on 14/12/2021.

## Background

Continuous deep sedation until death (CDSUD) of a patient is a procedure covered by the French Law of 2 February 2016 (Loi Claeys-Leonetti) creating new rights for patients with terminal illness [1]. While euthanasia and assisted suicide are still illegal in France, CDSUD is a response to refractory suffering (physical, psychological or existential) of the patient, who should be informed of this therapeutic option. Before implementing continuous deep sedation in a patient with a serious and incurable condition, and given the complexity and uniqueness of each situation, the physician has to follow 2 steps to respect both the wishes of the patient and the French law; These steps are: 1/ listening to, understanding and analyzing the patient’s request (with extensive consultation with the family, which is highly encouraged); 2/ verifying, through a collegial procedure, that the conditions established by French law are met, that the patient has the necessary capacity for judgment and that his/her request is made freely after he/she is provided with fair, clear and appropriate information [1]. In a patient who cannot express his/her wishes and if the physician stops life-sustaining treatment to avoid unreasonable obstinacy, the physician implements CDSUD unless the patient has objected to this in his/her advance directive [1]. Based on rare previous recent studies regarding CDSUD, we can state that a significant proportion of patients for who this process is administered are patients with cancer (e.g. in France: 81% [2], in Austria: 92% [3]).

Although these two requests do not always overlap, the literature suggests that whish for hastened death and whish for a CDSUD may present similarities. This distress, so difficult to delineate,has received multiple definitions [4]. It includes among others loss of personal meaning and purpose to life, fear of death, despair, anguish, hopelessness, a sense of burdening others, a sense of isolation, loss of dignity, helplessness, and betrayal [4, 5]. However, to the best of our knowledge, a description of the trajectory of the explicit whish for hastened death has only be empirically conducted once in France in a palliative care setting [6].

In 2018, 2 years after the promulgation of the Claeys-Leonetti law, the French National Center for Palliative care and End-of-Life (Centre National des Soins Palliatifs et de la Fin de Vie, CNSPFV) report mentioned that this procedure is not really integrated in the dedicated care structures yet, which of course testifies to the non-routine nature of the CDSUD, but also, perhaps, to the fact that the procedure is not clear to all, including patients and their relatives [7].

The death of a loved one is a stressful event for family caregivers even when death is expected, such as in the context of terminal phase of cancer. After the death of a patient with cancer, family caregivers can experience various poor physical and psychological outcomes including affective (e.g., anxiety and depression), cognitive (e.g., intrusive ruminations), behavioral (e.g., fatigue, agitation), and physiological-somatic outcomes (e.g., loss of appetite) [8, 9]. Up to 44% of bereaved individuals are at risk for a mental disorder [9] and this population is also more likely to report suicidal ideation than non-bereaved individuals with up to 31% of bereaved cancer caregivers reporting suicidal thoughts in the previous year [9]. Approximately 10–15% of individuals bereaved from natural deaths in adulthood experience Prolonged Grief Disorder (PGD). Prolonged grief has been associated with increased risk for anxiety, major depressive disorder and suicidal ideation [10]. The DSM 5-TR and the 11th International Classification of Diseases (ICD-11), classify PGD as its own nosological entity, although some research indicate that the ICD-11 criteria for a PGD diagnosis might be too lenient, but a good base for the detection of disturbed grief [11, 12].

The international and empirical literature on bereavement adjustment offers an integrative theoretical framework in order to highlight adaptive and maladaptive trends in grief process, as well as predictors of grief outcomes. This integrative framework [13–16] includes elements derived from the Stress and Coping Theory [17], the Attachment Theory [18, 19], The Freudian Grief Work Theory [20] and from Phenomenology [21] with regard to the meaning-making process that drive bereaved individuals. To date, some modifiable factors predicting mental health of bereaved relatives have been studied. Among them, burden of providing emotional and physical support to the cancer patient, perception of the patient’s death, satisfaction regarding end-of-life care, appear to be associated with the psychological adjustment of bereaved relatives in the cancer field [13, 21] Other factors, such as attachment patterns [22, 23] and anxiety and depression in the end-of –life period appear to be strongly associated with latter adjustment after the death of the patient [14, 22]. Psychological support for the distressed bereaved relatives is now highly recommended by international [24] and national health organizations (e.g. Plan Cancer 2014–2019). Although bereaved families were generally comfortable with the practice of CDSUD, some expressed a high level of emotional distress [25]. These findings confirmed the results of a systematic review that highlighted the little work on this issue [26].

The psychological adjustment of HCP and their experience of their job are becoming subjects of growing interest in scientific literature. Regarding the HCP, American studies indicated that palliative sedation (no specific focus on CDSUD) might arouse very strong ethical issues that can cause professional distress in nurses [27]. However, the nature of this distress is not systematically described. The majority of studies conducted in HCP are thus related to their representations and opinion related to this specific sedation [28, 29] and do not include French data. To our knowledge, only a recent French article [30] highlighted similar critical ethical tensions in medical and nursing teams regarding CDSUD; but the emotional experience of the professional was not specifically investigated. Considering this aspect could help to avoid professional turnover (which is a growing problem), and encourage their job satisfaction [31].

Debates are becoming increasingly heated about modifying the French law concerning the rights of patients at the end of life. Thus, it is critical to foster the development of bereavement research including new legal framework that may impact the grief experience in the cancer field. This longitudinal, mixed methods and multicentric study aims to explore with rigorous methods the adjustment of people in the heart of the CDSUD procedure and the very nature of their adjustment at mid-term.

## Methods/design

### Objectives of the study

In this perspective, the *primary objective* (PO) of the study is to describe the psychological adjustment and the experience of relatives and HCP of patients with cancer for whom a CDSUD is discussed (that is after the request of the patient or the decision of the medical team), using a mixed methods approach. The *secondary objectives* are: i/ to describe the trajectory of wish for a CDSUD (and its potential association with wish for hastened death) in patients who express a wish for a CDSUD (quantitative study). ii/ to highlight in relatives and professionals the potential factors associated with the psychological adjustment of these individuals (quantitative study). iii/ to capture the very nature of the grief process in the relatives of patients and their meaning-making process (qualitative study).

### Design and measures

The APSY-SED study is a prospective, longitudinal, multicenter, mixed-method study, designed to further understand the psychological adjustment of both relatives and HCP of patients who underwent a CDSUD procedure. Table 1 shows the various measures at the different assessment times [insert here].

**Table 1:** Measures for each population and the according time points

	Interview	BDI-II	STAI	RSQ	CANHELP	IES-R	ICG	PTGI	JCQ	Job satisfaction
Relatives										
• T1	X	X	X	X	X					
• T2		X	X			X	X	X		
• T3	X	X	X			X	X	X		
HCP										
• T1	X								X	X
• T2									X	X

### Time points:

- T1: after the discussion concerning CDSUD as an option and before the death of the patient. Patients, their relatives and their HCP are involved.
- T2: For HCP: 3 months after patient’s death; For relatives: 6 months after patient’s death.
- T3: For relatives only: 13 months after patients’ death. We choose to evaluate the outcomes in a time point of 13 months after CDSUD (over a 12 months’ time point) because we want to avoid an “anniversary” effect that could temporarily increase the intensity of the reactions.

Inclusions will be made over 18 months from January 2023.

### Outcomes:

#### Candidate covariates:

- For relatives: Attachment styles ; Satisfaction with end-of-life care ; Patient’s trajectory of wish for hastened death and wish for CDSUD.
- For HCP: sociodemographical variables (e.g. age, gender, seniority in the profession, position…).

#### Primary outcome:

Psychological adjustment respective to relatives and HCP’s situations.

- For relatives: Intensity of depression, anxiety, grief reactions; CDSUD-related distress, post-traumatic growth.
- For HCP: Job satisfaction and professional stress.

Regarding the patients, their *wish for CDSUD* and *potential wish for hastened death* will be collected by the professional caregivers and indicated to the psychologist in charge of data collection for the study. As requested by the law, professional caregivers have to regularly assess any evolution concerning request for CDSUD. Any evolution will then be noted by caregivers and transmitted to the researcher who will record this data. Medical and sociodemographic information will be collected in the medical files: demographic characteristics, diagnosis and localization of the neoplasia, presence of advances directives and of a surrogate, intervention of palliative care (in hospital and/or at home).

For relatives, starting T1, *Depression* symptoms will be evaluated through the Beck Depression Inventory (BDI-II, [32]): the French version of the BDI II is a 21-item self‐rated scale. It is a 4‐point Likert‐type scale ranging from 0 to 3 (overall score range: 0‐63). A score between 0 and 11 indicates a minimum level or absence of depression, between 12 and 19 a mild depression, between 20 and 27 a moderate depression and between 28 and 63 a severe depression. It is an instrument suitable for use in community screening and in studies with large samples, given that this scale is relatively short, self-administered, and has an easy scoring procedure. Results indicate that the BDI-II shows high reliability and internal consistency in several populations [33]. *Anxiety* will be evaluated with the State-Trait Anxiety Inventory (STAI): consisting of 2 subscales of 20 items each, to respectively measure states anxiety (S-anxiety) and trait anxiety (T-anxiety). Items were rated on a 4-point Likert scale (1 = almost never, 2 = sometimes, 3 = often, and 4 = almost always). The STAI score for each subscale (S-anxiety and T-anxiety) ranges from 20 to 80 for the 20 items, with higher scores suggesting greater anxiety. Both anxiety and depression will be reevaluated during T2 and T3 (see below). *Attachment* patterns will be indicated through the Relationship Scales Questionnaire (RSQ, French version, [34]), the most widely-used self-report concerning adult attachment. The RSQ consists of 30 items measured on a 5-point range scale ranging from 1 (not at all like me) to 5 (very much like me). Items 6, 9, and 28 are reverse-scored. According to Griffin & Bartholomew [35], the four attachment styles (i.e. Secure, Preoccupied, Dismissing and Fearful) can be derived by computing the mean rating of the items for each subscale. It shows high reliability and internal consistency in several populations [33]. *Satisfaction with palliative and End-of-life care* will be assessed through the CANHELP (Canadian Health Care Evaluation Project) questionnaire [36] designed to evaluate satisfaction with care for patients with advanced, life-limiting illnesses and their family caregivers in a hospital setting. The relatives’ version has 23 items and is validated in French. It is a self-administered questionnaire that assesses aspects of care delivery known to be important in end-of-life from families’ perspectives; an overall satisfaction score could be obtained as well as five domain scores (communication and decision-making, illness management, characteristics of doctors and nurses, relative’ involvement, and relationship with doctors). Sociodemographic information will also be collected (demographic characteristics, link with the patient with cancer [spouse / child / parent / sibling…], education level, employment status, mental health history, psychiatric treatment).

These questionnaires can be completed in paper format, by phone, or online via a secured Platform, according to the preferences of the participants.

A research interview will be conducted by a researcher with a curriculum in psychology at T1 after the implementation of CDSUD and before the death of the patient, with the volunteer relatives. We will trigger the discourse with the following non-directive question: “your relative is beneficiating from a CDSUD. Can we talk about what you are experiencing?”. This non-directive interview can cover the following topics: current emotional state, meaning and representations regarding potential CDSUD, perceived meaning of CDSUD in the patient according to the relatives. These interviews will allow the description of the subjective experience of the patients’ closed ones.

Then, during T2 and T3 several measurements will be submitted in order to assess psychological adjustment. First, *CDSUD-related stress/post-traumatic symptoms* will be assessed through the IES-R, a self-report measure of current subjective distress in response to a specific traumatic event [37]. The investigator can select this potential traumatic event: we assessed here the potential distressing impact of the CDSUD. This 22-item scale includes 3 subscales representative of the major symptom clusters of post-traumatic stress: intrusion, avoidance, and hyperarousal. Symptoms are rated on a 5-point Likert scale (0‐4), with higher scores indicating a high degree of distress. We classified participants as having a probable PTSD with IES‐R score > 33 [37]. *Intensity of grief reactions and the existence of Prolonged Grief Disorder* is going to be evaluated using the Inventory of Complicated Grief (ICG, [10; 38]). The ICG comprises 19 items, including yearning and preoccupation with the deceased, anger and bitterness, shock and disbelief, behavior change, including avoidance or proximity seeking. The respondents rated the degree to which the symptoms represented in the items applied to them in the last month on 5-point scales, which represent the frequency with which the symptoms occurred (ranging from almost never to always). The total score is calculated by summation of the item scores and gives an indication of the severity of grief. Scores ≥ 30 have been used as thresholds to identify clinically significant cases of Prolonged Grief Disorder [39, 40]. Disorder. Finally, the *Post-Traumatic Growth* Inventory [41] is an instrument for assessing positive outcomes reported by persons who have experienced traumatic events. This 21-item scale includes factors of New Possibilities, Relating to Others, Personal Strength, Spiritual Change, and Appreciation of Life. Items are rated on a 6-point scale ranging from 0 (“not at all”) to 5 (“very strongly”).

At T1, T2 and T3 the questionnaires could be mailed to the participants with stamped envelope to send back their questionnaires, completed by the phone, or completed online, according to their convenience.

A second research interview (face-to-face or by phone, based on the preferences of the participants) will be conducted at T3 (13 months after the death) by the same researcher, exploring in a non-directive way their grief experience (WP3).

For HCP, both professional stress and job satisfaction will be evaluated at T1 and T2 (3 months after patient’s passing). *Professional Stress and Professional experience* will be indicated with the Job Content Questionnaire (JCQ, “Karasek Questionnaire”, [42]). This 26-items self-questionnaire assesses 3 dimensions related to professional care: psychological demand (“job demand”), decisional latitude (i.e. autonomy in the professional activity, opportunity to be creative: “job control”) and social support of the colleagues and hierarchical superiors (“social support). Items of JCQ were scored on a four-point Likert-type scale, ranging from 1 = strongly disagree to 4 = strongly agree. This tool permits a comparison with reference data. From French data, the job strain threshold is set for a demands score higher than 20 and a control score lower than 71; the isostrain threshold is determined from a combining score of job strain and social support lower than 24 [33, 44]. *Job satisfaction* will also be investigated through the widely used one-item scale from Aiken et al. [44] ranging from 1 (very unsatisfied) to 4 (very satisfied). This item has been used to assess job satisfaction in previous studies [42, 45].

### Study setting

Participants will be recruited in 10 medical centers (oncology services and/or palliative care institutions and/or homecare services) belonging to the F.R.I.P.C Research Network. Furthermore, we plan to submit proposal for collaboration on the French National Platform for End-of-Life Research. Finally, invitations will be sent to all heads of units of palliative care institutions listed on the French Society for Palliative Care (SFAP) website.

The researchers involved in the recruitment (more precisely one Senior Researcher in Psychology and 2 Research Engineers with a curriculum in psychology) will phone twice a week identified referents in the recruitment centers in order to identify potential participants. The research engineers will also participate to weekly meetings in hospital services (nursing transmissions, medical meetings) in order to be aware of discussions regarding CDSUD. Finally, in the recruitment centers where this tool is implemented, the researchers will use text search software (e.g. at Gustave Roussy Hospital: Dr WareHouse©) in order to spot in the reports of the HCP the mention of a CDSUD.

Given the sensitive nature of the procedure, we plan to organize the information regarding the research as follow: one physician involved in the CDSUD procedure will briefly inform the patient (if he/she is able to express his will) and her/his relative(s) of the existence of a study devoted to the experience of CDSUD. The physician will inform them that a researcher will contact the relatives (by phone or face-to-face, according to their preferences) in order to state the nature and the objectives of the study. As soon as possible, the researcher will contact the potential participants and describe the study. Depending on what suits them, the researcher will either go to the medical institutions or call the participants. Questionnaires can be sent by post, completed online on a secured Platform, or can be completed over the phone (see [46]), and interviews conducted face-to-face or remotely according to the wishes of the participants. Participants will be informed of the longitudinal design of the study. Each recruitment will of course be recorded in each recruiting center, where researchers can contact participants in due time for follow-up. At T2 and T3, the participants will be contacted by telephone and mail to remind them of the continuation of the study. If they do not respond to 3 calls 5 days apart, after a month they will be considered out of the study. This protocol received the ethical approval of CSET at Gustave Roussy Hospital (CSET 2021/3334) and received ID-RCB N°:2021-A03042-39 on 14/12/2021. This ID-RCB number is delivered by the French National Agency for Medicines and Health Products Safety for all research carried out on human subjects [https://www.health-data-hub.fr/projets/apsy-sed-etude-de-lajustement-psychologique-dans-le-contexte-de-la-sedation-profonde-et].

### Participants

#### Inclusion criteria:

1/ Patient with cancer in palliative phase, whose impending death is expected within a few hours to a few days, age > 18, who requests for CDSUD or hastened death *OR* after a medical decision of a withdrawal of life-sustaining therapies has been made for a patient unable to express his/her willingness. 2/ the patient has at least one relative who is informed by the medical team and/or the patient of the option of CDSUD. Relative is defined as closed one of the patients and could be family members or friends. Several relatives of a same patient can be included in the study. 3/ the patient has at least one healthcare provider involved in the CDSUD who agrees to participate to the study. 4/ A collegial/multidisciplinary discussion underlined that CDSUD can be implemented for the patient according to the French law. 5/ For HCP who are involved in the CDSUD procedure, involvement is defined as medical prescription of CDSUD for physicians, daily nursing care for nurses and assistant nurses. HCP are here defined as physicians, nurses or assistant nurses. 6/ We collected the non-objection of the participants after reading the specific information letter.

#### Non-inclusion criteria:

1/Patients and or/relatives with a psychiatric disorder that alters the perception of the reality (as determined by the clinical staff). 2/Patients with severe cognitive impairment that hinders the participation to the study (as determined by the clinical staff). 3/Patients and/or relatives who do not have sufficient fluency in French to complete the questionnaire and to participate to the research interviews. 4/Patients whose death occurs before the effective implementation of the CDSUD. 5/Patients who requested a CDSUD but for whom this procedure was not effectively implemented.

### Efforts to address potential sources of bias

participants from various French regions, rural and urban, with various care offers (hospitalizations, home palliative care…), will be recruited, in order to have people (relatives and HCPs) representative of the population living in France. The (non)-inclusion criteria will also make it possible to constitute a sample that is both homogeneous in terms of experience of CDSUD (effective implementation of the procedure), but also with varied profiles. Finally, the different methods of administering questionnaires and interviews (paper/phone/online) should make it possible to broaden the recruitment of relatives. It will avoid a frequent bias when carrying out studies with bereaved people: that the most distressed people do not participate.

### Sample size justification

We plan to include 100 participants in the patients group; this number is based on previous data in palliative care research and corresponds to the reported mean cases of effectively implemented CDSUD in cancer care centers and palliative care institutions (≈ 10 cases/year in 10 centers of recruitment). We plan to include at least 150 participants in the relatives group (several relatives of the same patient will be recruited). Finally, we plan to include 50 professionals.

Regarding the qualitative part of the study, with IPA a saturation of the themes is not needed (because of the idiosyncratic nature of this method). We will propose research interviews to the volunteer relatives, and for analyses, we will constitute a purposive sample.

### Statistical analyses

#### Quantitative variables

Preliminary descriptive analyses will be performed in order to characterize the sample according to socio-demographical, clinical and psychological variables. Bivariate analyses will permit to describe the correlates of main outcomes (depression, anxiety, CDSUD-related stress, intensity of grief reactions, existence of a PGD, PTG at 13 months) in relatives and job strain and job satisfaction in HCP.

Multivariate models for the main outcomes of psychological adjustment will use regression models to examine relationships between all predictors jointly and outcomes. Regression models will be hierarchical. To test the predictors of the existence of a Prolonged grief disorder (categorical variable), we will use logistic regression. Analyses will be performed on standardized measures. Analyses of variance (ANOVA) will be used in order to consider the role of predictors in the total variance of the dependent variables. More precisely, it will allow us to describe the respective influence of CDSUD characteristics versus personal characteristics of relatives on dependent variables.

Principal Components Analyses (PCA) will be conducted in order to test associations of variables jointly, considering the total variance of the selected variables. Kaiser’s criterion [47] will be used as a threshold to keep the main components of the PCA (i.e., eigenvalue _ 1). Then, scores of depression (BDI-II), anxiety (STAI), CDSUD-induced stress (IES-R), grief reactions (ICG) post-traumatic growth (PTGI), attachment styles (RSQ), satisfaction with care (CANHELP) will be included in the PCA in order to be grouped in dimensions (or principal components).

All analyses will be conducted using SPSS 25.

#### Qualitative analysis

Interviews, audio-recorded and transcribed verbatim, will be subjected to Interpretative Phenomenological Analysis (IPA). This method was chosen to understand the complex system of meanings attached to a unique, subjective and eminently intimate phenomenon [48]. This method relies on a double hermeneutic wherein the researcher attempts to investigate the way the participant makes sense of his/her own subjective experience [48–50] A standardized procedure ensures the methodological rigor [51]. First, every interview will be read and the main themes will be coded by a first researcher. Discourse themes will be identified (sufficiently characteristic topics, implying that the researcher can see a common and stable sense in them). The connections between the themes will then be studied and the major themes will be identified. Lastly, an interpretative account will be produced that highlights and analyses the experience through experiential themes, by illustrating the discourse. Throughout this study, we will follow the COREQ guidelines for qualitative research [52].

## Discussion

Given the recency of the French law organizing CDSUD, our study aims to add both quantitative and qualitative data on variables not often investigated regarding this procedure. The strengths of it lie in the number of prospected participants, with most of the stakeholders (patients, their relatives and their HCP) being included. This would hopefully allow having a global perspective on the experience of most individuals involved in CDSUD. The mixed-method will help to understand the complexity of their experience: interviews will add helpful information in complement of quantitative data. Our goal to recruit participants across the country will allow for maximum representation of people being exposed to CDSUD in France.

Possible limitations can be pinpointed, with the focus of CDSUD being on cancer care. Therefore, the specificities of CDSUD in other palliative situations (e.g. for other terminal illnesses) may not be represented. Furthermore, we acknowledge that the reality of CDSUD in France may vary greatly from other countries, since the country’s legislation is considered by some to be “The French exception” [53]. Finally, the broad inclusion of relatives and HCP, with no distinction between family or friends, or between the different HCP, may not be able to represent the unique experience of specific relatives or HCP. Therefore, our ambition with this prospective research is to open the door to further findings regarding CDSUD in France.

Our findings will allow to describe the experience of caregivers confronted with CDSUD and then to elaborate and refine interventions fostering their well-being. Empirical findings on CDSUD among patients with cancer in France could be compared with existing data in other countries and with results related to other medical fields where CDSUD is also conducted (e.g. HIV, ALS…). Our investigation of the grief process and of the possible occurrence of PGD for relatives may help us create tailored psychological interventions that consider the specificities of grief after a CDSUD procedure, while being based on the existing literature on treatment of complicated grief [54–56].

## Data Availability

The dataset supporting this article will be available in the Health Data Hub repository, [N° F20220121190541; https://www.health-data-hub.fr/projets/apsy-sed-etude-de-lajustement-psychologique-dans-le-contexte-de-la-sedation-profonde-et]

## Abbreviations

CDSUD: Continuous deep sedation until death
CNSPFV: Centre National des Soins Palliatifs et de la Fin de Vie
HCP: Healthcare provider
ICD: International Classification of Diseases
IPA: Interpretative Phenomenological Analysis
PCA: Principal Components Analyses
PGD: Prolonged Grief Disorder
PTG: Post-traumatic growth
PTSD: Post-traumatic Stress Disorder
